# Significance of levothyroxine treatment on serum lipid in pregnant women with subclinical hypothyroidism

**DOI:** 10.1186/s12884-022-04950-2

**Published:** 2022-08-06

**Authors:** Yuxi Yang, Huabing Yuan, Xueran Wang, Zheng Zhang, Ruixia Liu, Chenghong Yin

**Affiliations:** 1 Department of Internal Medicine, Beijing Obstetrics and Gynecology Hospital, Capital Medical University, Beijing Maternal and Child Health Care Hospital, Beijing, 100026 China; 2 Department of Cardiovascular Medicine, Hebei General Hospital, Shijiazhuang, 050051 Hebei China; 3 Department of Central Laboratory, Beijing Obstetrics and Gynecology Hospital, Capital Medical University, Beijing Maternal and Child Health Care Hospital, Beijing, 100026 China

**Keywords:** Levothyroxine treatment, Serum lipid, Subclinical hypothyroidism

## Abstract

**Background:**

There is no consensus reference range for serum lipid levels during pregnancy. The benefit of levothyroxine (L-T4) on serum lipid levels are unclear among pregnant women with subclinical hypothyroidism (SCH).

**Objective:**

To determine the recommended reference ranges for serum lipid concentrations during pregnancy and effects of L-T4 treatment on serum lipids in pregnant women with SCH.

**Design:**

Cohort study.

**Methods:**

A analysis of 20,365 women in the first trimester was conducted at Beijing Obstetrics and Gynecology Hospital, Capital Medical University during 2018–2020. After excluding women with adverse pregnancy outcomes, we determined the reference range of serum lipid in the first and third trimesters of pregnancy by using median and quartile to determine appropriate percentiles. Next, we divided into three groups as follows: SCH L-T4 treatment group (*n* = 319), SCH non-intervention group (*n* = 103) and the control group(*n* = 9598).

**Results:**

The recommended reference range for serum lipids in the first trimester of pregnancy should be: TC < 5.33 mmol/L, TG < 1.73 mmol/L, LDL-C < 3.12 mmol/L and HDL-C > 1.1 mmol/L, and in third trimester of pregnancy should be: TC < 8.47 mmol/L, TG < 4.86 mmol/L, LDL-C < 5.3 mmol/L and HDL-C > 1.34 mmol/L.

There are significant differences in TC and LDL-C levels between SCH treatment group and SCH non-intervention Group (*P* = 0.043, *P* = 0.046; respectively).

**Conclusions:**

We determine the recommended reference ranges for serum lipid concentrations during pregnancy. TC and LDL-C levels in pregnant women with SCH could improve after L-T4 treatment.

**Supplementary Information:**

The online version contains supplementary material available at 10.1186/s12884-022-04950-2.

## Introduction

Subclinical hypothyroidism (SCH) is defined as the co-presentation of a thyroid-stimulating hormone (TSH) level above the normal range (4.0–10.0 mIU/L) and a serum free thyroxine (FT4) level within the normal range. It is common among women of childbearing age and because of its asymptomatic nature and insidious clinical symptoms, it can be easily missed in clinical practice. The prevalence of SCH during pregnancy is approximately 2%–3% [1]. SCH usually linked to some adverse pregnancy outcomes, such as preterm birth, miscarriage, gestational diabetes mellitus (GDM), and low birth weight [2–5].

Elevated TSH concentrations in SCH may alter the synthesis and degradation of lipids as well as the function of various enzymes in the lipid metabolism pathway [6]. Key effects of elevated TSH include down-regulation of low-density lipoprotein cholesterol (LDL-C) receptor expression and increased proprotein convertase subtilsin-kexin type 9 (PCSK-9), which together result in higher total cholesterol (TC), LDL-C, and triglyceride (TG), and lower high-density lipoprotein cholesterol (HDL-C) [7–9]. Previous study has indicated that non-gestational SCH leads to elevated blood lipid levels with TSH concentrations elevation [10]. some conditions such as hyperemesis gravidarum and adiposity which may affect serum lipids and TSH levels. Teixeira et al. [11] found TSH levels have effects on adiposity, which in turn may affect thyroid function. Additionally, serum TSH levels elevated due to adiposity. Similarly, a large retrospective observational study of 949 patients with normal thyroid function and severe obesity showed that bariatric surgery promotes a decrease of TSH levels [12]. In addition to adiposity, hyperemesis gravidarum is associated with lipid peroxidation [13]. Studies had also shown that L-T4 treatment of SCH can improve serum lipid levels, while the research object were all adults, not pregnant women [14–16].

However, there is no consensus reference range for maternal TC, TG, LDL-C, HDL-C levels during gestation. Given the lack of trimester-specific reference values cut-off points, the aim of the present cohort study was to discuss recommended reference ranges for serum lipid concentrations in the first and third trimesters of pregnancy and effects of L-T4 treatment on serum lipid levels in pregnant women with SCH.

## Material and methods

### Study design and participants

This cohort study recruited pregnant women in their first trimester who visited the Beijing Obstetrics and Gynecology Hospital, Capital Medical University between January 2018 and May 2020. All participants answered a questionnaire during early pregnancy(6–13^+6^ weeks of gestation) about demographic and obstetric characteristics (age, weight, parity, and history of adverse pregnancy outcomes), history of thyroid disease before pregnancy, history of hypertension, and history of diabetes. Written consent has been obtained from each patient or subject after full explanation of the purpose and nature of all procedures used. We continued to follow up these women through the middle and third trimesters of pregnancy until delivery. A total of 20,365 women who had complete thyroid examination data and lipid data in the first and third trimesters of pregnancy( 6–13^+6^ weeks of gestation and 28–33^+6^ weeks of gestation) respectively were enrolled in this study.

**Fig. 1:**
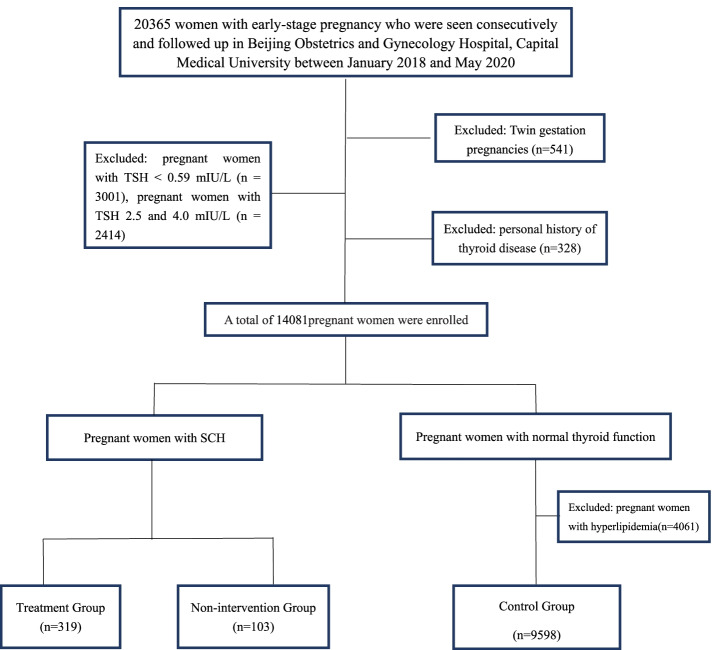
Flow chart of patient inclusion

We excluded pregnant women with TSH levels < 0.59 mIU/L (*n* = 3001), mild TSH elevation(TSH concentration between 2.5 and 4.0 mIU/L, *n* = 2414) and those who had thyroid disease before pregnancy (including thyroid cancer, thyroid nodule, and Hashimoto thyroiditis; *n* = 328). When all the women had given birth, we also excluded those who had twin or multiple pregnancies (*n* = 541). Thus, ultimately, 14,081 pregnant women were included in this cohort study (Fig. 1).

We grouped the women as follows: SCH treatment group (treatment begin at 6–13^+6^ weeks of gestation, *n* = 319), SCH non-intervention group (*n* = 103) and normal thyroid function group (*n* = 13,659).

### Ethical considerations

Our study protocol was approved by the Ethics Committee of Beijing Obstetrics and Gynecology Hospital, Capital Medical University (Review approval number: 2018-KY-003–01).

### Definitions

SCH was defined as a normal FT4 level with TSH elevation (4.0–10 mIU/L). The reference values for the normal FT4 range during the first trimester were 11.8–18.4 pmol/L. Enzyme immunoassay were used to measure the serum FT4 and TSH concentrations. The methods and kits used for the FT4 and TSH tests in our hospital remained the same throughout the study period.

### Serum lipids measurement

All pregnant women collected venous blood samples after overnight fasting. The concentrations of TC, TG, HDL-C and LDL-C were determined for each sample. Automatic biochemical analyser (AU5400, Beckman, US) were used to measure TC, TG, HDL-C and LDL-C detection kits (Beckman, US). All measurements were measured using continuous monitoring methods with appropriate quality control prior to measurement.

### Adverse pregnancy outcomes

Adverse pregnancy outcomes included preterm delivery (defined as birth before 37 weeks of gestation),, gestational diabetes mellitus (screened for at 24–28 weeks, and diagnosed according to the criteria of the International Association of Diabetes and Pregnancy Study Groups, which require a 75-g oral glucose tolerance test and cut-off values of > 5.1 mmol/L, > 10.0 mmol/L, and > 8.5 mmol/L for fasting blood glucose, blood glucose at 1 h after sugar intake, and blood glucose at 2 h after sugar intake, respectively), gestational hypertension and macrosomia (≥ 4000 g).

### Statistical analysis

All statistical analyses were performed using the SPSS, version 24.0 (SPSS Inc., Chicago, IL, USA). In order to observe the normal trend of serum lipid levels during the first and third trimesters of pregnancy, we excluded those pregnant women with overweight (BMI is between 24.0 and 27.9 kg/m^2^)/obesity (BMI ≥ 28.0 kg/m^2^), gestational hypertension, GDM, preterm birth and macrosomia. Then, using appropriate percentiles and median to descriptive analyses of TC, TG, LDL-C and HDL-C levels for this group (excluded adverse pregnancy outcomes) in first and third trimester of pregnancy. Finally, to establish the lipid reference ranges suitable for the first and third trimesters of pregnancy. Categorical variables were presented as frequency (percentage), and continuous variables were presented as mean (standard deviation [SD]) or median (interquartile range), as appropriate. Analysis of variance was used for normally distributed continuous variables in the multiple subgroup analyses. The Kruskal–Wallis test was used to evaluate continuous variables with non-normal distributions. To compare categorical variables, we used the chi square test or Fisher exact test. The Bonferroni correction was applied for pairwise comparisons. To evaluate the effect of L-T4 treatment on SCH with hyperlipidemia, we used generalized estimating equation. Stratified analysis by different BMI groups was employed to further compare treatment and level of serum lipids. Multivariate logistic regression model was used to evaluate interaction effect between BMI and treatment on the level of serum lipids. A *P* value < 0.05 or a *P* value < 0.05/n in the Bonferroni corrections was considered statistically significant.

## Results

### Reference range of serum lipid in the first and third trimesters of pregnancy

In total, 20,365 pregnant women were included in our study. After excluded GDM (*n* = 3038), overweight/obesity (*n* = 2040), gestational hypertension (*n* = 806), preterm birth (*n* = 867), macrosomia (*n* = 1305), 12,309 pregnant women remained. Table 1 presents the median (interquartile range) concentrations and appropriate percentiles for TC, TG, LDL-C and HDL-C in both first and third trimesters of pregnancy. Serum concentrations of TC, TG, LDL-C and HDL-C in the third trimester were significantly higher than those in the first trimester of pregnancy, especially the level of TG increased 2.81 times in the third trimester of pregnancy. The level of TC, TG, LDL-C and HDL-C had 1.57, 2.81, 1.70 and 1.22-fold elevations from the first to third trimester, respectively. Recommended reference range for serum TC, TG and LDL-C in the first and third trimesters of pregnancy were less than the 95th percentile and that of HDL-C were greater than the 5th percentile [17]. Specifically, the recommended reference range for serum lipids in the first trimester of pregnancy should be: TC < 5.33 mmol/L, TG < 1.73 mmol/L, LDL-C < 3.12 mmol/L and HDL-C > 1.1 mmol/L, and in third trimester of pregnancy should be: TC < 8.47 mmol/L, TG < 4.86 mmol/L, LDL-C < 5.3 mmol/L and HDL-C > 1.34 mmol/L.

**Table 1:** Serum lipid levels of pregnant women in the first and third trimesters of pregnancy

Serum lipid levels	Trimester	median (interquartile range)	Percentile (*n* = 12,309)								
			2.5%	5%	10%	25%	50%	75%	90%	95%	97.5%
TC (mmol/L)	First	4.11(3.72–4.56)	3.03	3.19	3.38	3.72	4.11	4.56	5.02	5.33	5.64
	Third	6.46(5.78–7.22)	4.58	4.85	5.20	5.78	6.46	7.22	7.98	8.47	8.92
TG (mmol/L)	First	0.92(0.73–1.17)	0.50	0.54	0.61	0.73	0.92	1.17	1.49	1.73	2.00
	Third	2.76(2.25–3.44)	1.54	1.68	1.88	2.25	2.76	3.44	4.23	4.87	5.57
LDL-C (mmol/L)	First	2.11(1.77–2.48)	1.20	1.35	1.50	1.77	2.11	2.48	2.85	3.12	3.41
	Third	3.54(2.91–4.20)	1.85	2.09	2.39	2.91	3.54	4.20	4.86	5.30	5.72
HDL-C(mmol/L)	First	1.51(1.34–1.71)	1.03	1.10	1.19	1.34	1.51	1.71	1.91	2.04	2.16
	Third	1.85(1.62–2.09)	1.25	1.34	1.44	1.62	1.85	2.09	2.34	2.50	2.66

*TC* Total cholesterol, *TG* Triglycerides, *LDL-C* Low-density lipid cholesterol, *HDL-C* High-density lipid cholesterol

### Baseline characteristics of SCH pregnant women and control group

The baseline characteristics of the patients in the L-T4 treatment group (*n* = 319), non-intervention group (*n* = 103) and the control group are shown in Table 2. Control group was defined as normal thyroid function and serum lipid(*n* = 9598).

**Table 2:** Characteristics of pregnant women with SCH who did or did not receive treatment and control subjects

Characteristic	SCH non-intervention Group (*n* = 103)	SCH treatment Group (*n* = 319)	Control group (*n* = 9598)	*P* value
Age (years)	31.84(3.83)	30(28–33)	30(28–33)	0.014
BMI (kg/m^2^)	22.70(3.77)	21.56(19.61–24.01)	21.48(19.83–23.44)	0.115
TSH (mIU/L)	4.56(4.22–5.22)	4.75(4.33–5.59)	1.34(0.96–1.79)	< 0.001
FT4 (pmol/L)	14.73(1.43)	14.76(1.47)	15.92(14.77–16.91)	< 0.001
GA at delivery (wks)	39 (38–40)	38 (38–40)	39 (38–40)	0.695
Neonatal weight (g)	3349.08(399.47)	3307.07(483.43)	3350 (3085–3620)	0.249
History of adverse pregnancy outcomes, n (%)	3 (2.91)	3 (0.94)	159(1.66)	0.294
History of hypertension, n (%)	1 (0.97)	3(0.94)	40(0.42)	0.532
History of diabetes, n (%)	0	0	31 (0.32)	
Cesarean section rate, n (%)	41 (39.81)	117(36.68)	3374(35.15)	0.154

*TSH* Thyroid-stimulating hormone, *FT4* Free thyroxine, *GA* Gestational age, *BMI* Body mass index

The Kruskal–Wallis test was used to compare non-normally distributed continuous variables. The chi-square test or Fisher exact test was used to compare categorical variables among the three groups

Of all pregnant women with SCH, 348(82.5%) were tested for TSH concentration during the second trimester(20–23^+6^ weeks of gestation) and 304(72.0%) were tested for TSH concentration during the third trimester(28–33^+6^ weeks of gestation). No significant differences in BMI, gestational age (GA) at delivery, neonatal birth weight, history of adverse pregnancy outcomes, histories of hypertension, and rate of cesarean section were found among the three groups. The median (interquartile range) age significantly differed between L-T4 treatment group (30 years; 28–33 years), non-intervention group (31.84 years; 3.83), and the control group (30 years; 28-33 years; *P* = 0.014). The TSH concentration was significantly higher in the L-T4 treatment group and non-intervention group than in the control group (*P* < 0.001). In contrast, the FT4 value was significantly higher in the control group than in the other two groups (*P* < 0.001), though the FT4 value was within the normal range in all three groups.

### Serum lipid levels of SCH pregnant women after L-T4 treatment

Serum concentrations of TC, TG, LDL-C and HDL-C in SCH pregnant women with hyperlipidemia treatment group(*n* = 319) and non-intervention group (*n* = 103) in the first and third trimester of pregnancy are shown in Table 3. Table 4 shows the effects of L-T4 on pregnant women with SCH who did or did not receive treatment. There are significant differences in TC and LDL-C levels between SCH treatment group and SCH non-intervention Group (*P* = 0.017, *P* = 0.011; respectively), which indicated that L-T4 treatment could reduce TC and LDL-C levels in pregnant women with SCH. No significant difference in TG and HDL-C concentration between pregnant women with SCH and non-intervention group after L-T4 treatment (*P* > 0.05). L-T4 treatment had a significant interaction effect with age and BMI. Treatment could not directly reduce TG level, but the interaction effect between treatment and age reduced TG level (*P* = 0.022). Identically, treatment could not directly reduce TG and HDL-C levels, but the interaction effect between treatment and BMI reduced TG and HDL-C levels(*P* < 0.001). There are no significant interaction in L-T4 treatment and TSH, FT4 levels(*P* > 0.05). When dividing BMI into normal weight, overweight and obesity, obesity can aggravate the effect of treatment on HDL-C levels, although they do not promote treatment (OR:0.07; 95% confidence interval: 0.10–0.38, *P* < 0.001) (Supplement 1).

**Table 3:** Serum lipid levels of pregnant women with SCH who did or did not receive treatment

Serum lipid levels		**SCH non-intervention Group (*n* = 103)**	**SCH treatment Group (*n* = 319)**
TC (mmol/L)	first trimester of pregnancy	4.37(0.77)	4.29(3.80–4.81)
	third trimester of pregnancy	6.61(1.29)	6.42(5.62–7.05)
TG (mmol/L)	first trimester of pregnancy	1.06(0.85–1.47)	1.07(0.81–1.44)
	third trimester of pregnancy	2.99(2.49–3.74)	2.81(2.32–3.52)
LDL-C(mmol/L)	first trimester of pregnancy	2.28(0.61)	2.20(1.77–2.61)
	third trimester of pregnancy	3.64(1.10)	3.41(2.70–4.09)
HDL-C(mmol/L)	first trimester of pregnancy	1.55(1.34–1.77)	1.55(1.35–1.75)
	third trimester of pregnancy	1.80(1.61–1.97)	1.84(0.36)

*TC* Total cholesterol, *TG* Triglycerides, *LDL-C* Low-density lipid cholesterol, *HDL-C* High-density lipid cholesterol

continuous variables were presented as mean (standard deviation [SD]) or median (interquartile range)

**Table 4:** Effects of L-T4 on pregnant women with SCH who did or did not receive treatment

Serum lipid levels	group		group*age		group*BMI		group*TSH		group*TSH	
	wald x^2^	*P* value	wald x^2^	*P* value	wald x^2^	*P* value	wald x^2^	*P* value	wald x^2^	*P* value
TC (mmol/L)	5.700	0.017	10.233	0.006	2.627	0.269	1.127	0.569	2.420	0.298
TG (mmol/L)	0.274	0.600	7.608	.022	21.548	< 0.001	2.192	0.334	0.519	0.771
HDL-C (mmol/L)	2.466	0.116	1.227	0.541	28.795	< 0.001	0.164	0.921	1.616	0.771
LDL-C (mmol/L)	6.430	0.011	5.800	0.055	0.115	0.944	0.020	0.990	2.816	0.245

*TC* Total cholesterol, *TG* Triglycerides, *LDL-C* Low-density lipid cholesterol, *HDL-C* High-density lipid cholesterol

The generalized estimating equation was used to compare categorical variables among the two groups

Dependent variable: TC, TG, HDL-C and LDL-C

Model: (intercept), group, group * age, group * BMI, group * TSH, group * FT4

## Discussion

The current cohort study was performed to evaluate the recommended reference ranges for serum lipid concentrations in the first and third trimesters of pregnancy and effects of L-T4 treatment on serum lipid levels in pregnant women with SCH. Wang et al. [17] recommend that the reference values of serum TC, TG and LDL-C in early and middle pregnancy should be less than the 95th percentiles and the reference value of HDL-C should be greater than the 5th percentile. So using the same method, we found that the recommended reference range for serum lipids in the first and third trimesters of pregnancy of pregnancy, which is appropriated for Beijing area. We also found that L-T4 treatment reduced the level of TC and LDL-C in SCH pregnant women.

To date, there are no unified reference ranges evaluate serum lipid in women during pregnancy, our recommended reference range of serum lipid during the various periods of pregnancy is suitable for pregnant women in Beijing area. Furthermore, we also found serum concentrations of TC, TG, LDL-C and HDL-C were significantly higher in pregnant women in the third trimester than they were in the first trimester, especially 2.81-fold TG elevation in third trimester of pregnancy. Previous studies revealed that from the 12^th^ week of pregnancy, TC, TG, LDL-C and HDL-C gradually increase, especially in the second and third trimesters, which was consistent with our study [18–23]. Determining reference range for serum lipids may predicting possible adverse pregnant outcomes. A population-based study for 934 pairs of mothers and neonates showed that high TG during late pregnancy was independently associated with increased risk of GDM, preeclampsia and macrosomia [24]. A prospective study by Adank *et al* [25]*.* found that TG level in the first trimester of pregnancy could increase the of risk of large-for-gestational age (LGA). Similar to their results, Lu et al. [26] demonstrated that TG level in the first trimester of pregnancy had a strong positive association with GDM. Therefore, with the establishment of reference ranges for serum lipids during pregnancy, the level of serum lipids can be used as predictive and warning factors for some adverse pregnancy outcomes.

The main finding of our study was that pregnant women with SCH who received L-T4 treatment could improve TC and LDL-C levels compared to non-intervention group (*P* = 0.043, *P* = 0.046; respectively). Our further results have clarified the interaction effect between treatment and BMI, it was the first time to compare the L-T4 treatment on TG and HDL-C levels between the different BMI groups. We found that treatment was only associated with lower HDL-C levels in the case of obesity, obesity could aggravate the effect of treatment on HDL-C levels, although they do not promote treatment. This was the innovation point of our study, which has important clinical significance.

As is well-known, SCH patients often present lipid abnormalities, especially elevated TC and LDL-C [27]. Amsterdam Born Children and Their Development cohort study reported that high TG in late pregnancy was independently associated with increased risk of GDM, preeclampsia and premature delivery [27, 28]. Thus, the control of serum lipids may benefit SCH patients. However, pregnant women shall not take statins during pregnancy to reduce serum lipid. Most current studies have focused on the reduction of serum lipid in adults with SCH after L-T4 treatment [29]. To date, clinical trials have not consistently shown the benefit of L-T4 treatment in improving lipid levels in SCH patients. Kong et al*.* [30] found that L-T4 treatment had no effect on serum lipids and other metabolic indicators in a 6-month randomized trial of female with SCH. Our study is the first in China to report effects of L-T4 therapy on serum lipid with SCH pregnant women.

### Strengths and limitations of this study

A strength of this study is that all study participants completed a comprehensive questionnaire, which included a detailed review of their medical history, serum lipid tests (including TC, TG, LDL-C and HDL-C) and the results of thyroid function tests (including T4and TSH). We also continued to follow up the patients through the middle and third trimesters of pregnancy until delivery.

Nevertheless, our study has several limitations. The main limitation of our study was that the study population was small for some subgroups. Therefore, prospective collection of data from multiple centers is needed to verify the results of this study. In addition, our study was a non-randomized controlled trial, and the quality of evidence for the results was not as good as that of a randomized controlled trial.

In conclusion, the levels of TC, TG, LDL-C and HDL-C have been changing dynamically during pregnancy. Therefore, we determine the recommended reference ranges for serum lipid concentrations in the first and third trimesters of pregnancy. Furthermore, we found that L-T4 treatment could reduce TC and LDL-C levels in pregnant women with SCH. These findings may provide more evidence to support lipid screening during pregnancy, further guidance on the treatment of thyroid disease in pregnant women and inform the development of future guidelines.

### Trial registration

Chinese Clinical Trial Register, ChiCTR2100047394. Registered 16 June 2021—Retrospectively registered, http://www.chictr.org.cn.

## Supplementary Information

**Additional file 1: Table S1.** Multivariate logistic regression among different BMI groups.

## Data Availability

The datasets used and/or analysed during the current study available from the corresponding author on reasonable request.
