# Supplementary material for: Significance of levothyroxine treatment on serum lipid in pregnant women with subclinical hypothyroidism
Source: BMC Pregnancy Childbirth. 2022 Aug 6;22:623. doi: 10.1186/s12884-022-04950-2 (PMC9356505; doi:10.1186/s12884-022-04950-2)
Supplement: Supplementary file 1 — Additional file 1: Table S1. Multivariate logistic regression among different BMI groups. [file 12884_2022_4950_MOESM1_ESM.docx]

**Table 1.** Multivariate logistic regression among different BMI groups

| **Dependent variable** | **Independent variables** | **OR (95%CI)** | ***P* value** |
| --- | --- | --- | --- |
| TG (mmol/L) | BMI (1) | 0.20(0.05-0.74) | 0.086 |
|  | BMI (2) | 0.28(-0.22-0.89) | 0.232 |
|  | BMI (3) | 0.32(-0.62-0.63) | 0.983 |
| HDL-C (mmol/L) | BMI (1) | 0.05(-0.19-0.01) | 0.071 |
|  | BMI (2) | 0.07(0.10-0.38) | 0.001 |
|  | BMI (3) | 0.08(-0.01-0.31) | 0.072 |

Abbreviation: BMI: body mass index

BMI (1): overweight vs. normal weight; BMI (2): obesity vs. normal weight; BMI (3): overweight vs. obesity
